# In Vitro Assessment of Bio-Functional Properties from *Lactiplantibacillus plantarum* Strains

**DOI:** 10.3390/cimb44050158

**Published:** 2022-05-19

**Authors:** Francesco Letizia, Gianluca Albanese, Bruno Testa, Franca Vergalito, Diletta Bagnoli, Catello Di Martino, Petronia Carillo, Lucia Verrillo, Mariantonietta Succi, Elena Sorrentino, Raffaele Coppola, Patrizio Tremonte, Silvia Jane Lombardi, Roberto Di Marco, Massimo Iorizzo

**Affiliations:** 1 Department of Agriculture, Environmental and Food Sciences, University of Molise, Via De Sanctis, 86100 Campobasso, Italy; f.letizia@studenti.unimol.it (F.L.); g.albanese@studenti.unimol.it (G.A.); bruno.testa@unimol.it (B.T.); franca.vergalito@unimol.it (F.V.); d.bagnoli@studenti.unimol.it (D.B.); lello.dimartino@unimol.it (C.D.M.); succi@unimol.it (M.S.); coppola@unimol.it (R.C.); tremonte@unimol.it (P.T.); silvia.lombardi@unimol.it (S.J.L.); 2 Department of Environmental Biological and Pharmaceutical Sciences and Technologies, University of Campania “Luigi Vanvitelli”, Via Vivaldi 43, 81100 Caserta, Italy; petronia.carillo@unicampania.it; 3 Institute of Genetics and Biophysics “Adriano Buzzati-Traverso”, National Research Council (CNR), 80131 Naples, Italy; lucia.verrillo@igb.cnr.it; 4 Department of Medicine and Health Science “V. Tiberio”, Università degli Studi del Molise, 86100 Campobasso, Italy; roberto.dimarco@unimol.it

**Keywords:** antioxidant activity, exopolysaccharides, *Lactiplantibacillus plantarum*, probiotic, β-glucosidase, γ-aminobutyric acid

## Abstract

In recent years, alongside the conventional screening procedures for the evaluation of probiotics for human usage, the pharmaceutical and food industries have encouraged scientific research towards the selection of new probiotic bacterial strains with particular functional features. Therefore, this study intended to explore novel functional properties of five *Lactiplantibacillus plantarum* strains isolated from bee bread. Specifically, antioxidant, antimicrobial and β-glucosidase activities, exopolysaccharides (EPS) production and the ability to synthesize γ-aminobutyric acid (GABA) were evaluated. The results demonstrated that the investigated *L. plantarum* strains were effective in inhibiting the growth of some human opportunistic pathogens in vitro (*Pseudomonas aeruginosa*, *Escherichia coli*, *Proteus mirabilis*, *Enterococcus faecalis* and *Staphylococcus aureus*). Moreover, the evaluation of antioxidant and β-glucosidase activity and of EPS and GABA production, revealed a different behavior among the strains, testifying how these properties are strongly strain-dependent. This suggests that a careful selection within a given species is important in order to identify appropriate strains for specific biotechnological applications. The results highlighted that the five strains of *L. plantarum* are promising candidates for application as dietary supplements in the human diet and as microbial cultures in specific food productions.

## 1. Introduction

The term ‘probiotic’ has been widely debated over the years, prompting a change in the generally accepted definition [1]. The current definition, formulated in 2002 by an FAO (Food and Agriculture Organization of the United Nations) and WHO (World Health Organization) working group of experts, states that probiotics are “live strains of strictly selected microorganisms which, when administered in adequate amounts, confer a health benefit on the host” [2]. This description was deemed relevant, and therefore maintained, by the Scientific Association for Probiotics and Prebiotics (ISAPP) in 2013 [3].

Besides the literal meaning, some of the criteria for microorganisms selection must be considered before their applicability as probiotics in the human diet: a probiotic agent must show non-pathogenic properties; ability to survive in the digestive tract; adherence to the intestinal epithelium; colonization of the intestinal tract; production of antimicrobial compounds; adequate survival (stability) in the form of powder, liquid or food [3,4,5]. In addition to these primary characteristics, there are further functional attributes that can be contemplated in the selection of new probiotics [6,7,8,9].

Specifically, the selection of lactic acid bacteria (LAB) as probiotics possessing β-glucosidase activity and also capable of producing exopolysaccharides (EPSs) and γ-aminobutyric acid (GABA) has received much attention in recent years [10,11,12,13,14]. GABA (IUPAC name: 4-aminobutanoic acid) is a non-protein ubiquitous amino acid that is biosynthesized via the α-decarboxylation of glutamate, by glutamate decarboxylase (GAD) [15]. The pharmaceutical and food industries have been performing extensive studies to develop GABA-rich dietary supplements and fermented foods aiming to leverage the manifold health benefits of this amino acid, including gut modulation, neurostimulation, and cardioprotection [10,16].

About the β-glucosidase activity of LABs, it is of particular interest since it is able to increase the bioavailability of polyphenols. Several studies indicate the potential effect of polyphenols such as flavonoids and anthocyanins in reducing the incidence of cardiovascular disease, cancer, hyperlipidemias and other chronic diseases through the intake of anthocyanin-rich foods [17]. However, the effectiveness strongly depends on their bioavailability [18]. Anthocyanins contained in fruit and vegetables are bound to one or more glycosidic units. The biological activity of anthocyanins can be impaired by glycosylation, which increases their solubility in water, but on the other hand, it also makes them less reactive to free radicals and metals, thus reducing their antioxidant activity [19]. The glycosidase activity of several LABs removes the glycoside and this hydrolysis increases the bioavailability and intestinal absorption of flavonoids, which is essential for their health benefits [20]. Moreover, several LABs are also capable of producing capsular or extracellular polysaccharides (EPS), with desirable technological properties and biological activities [13,21]. EPS are widely used as viscous agents, stabilizers and emulsifiers in the food industry [22,23,24,25]. In addition, the EPS produced by LABs may be important for the survival of probiotics during gastrointestinal transit and adhesion to the intestinal wall, as well as being known to exhibit several prebiotic properties [21,22,26].

Among LABs, *Lactiplantibacillus plantarum* (formerly *Lactobacillus plantarum*) [27] is a versatile bacterium with high adaptability to many different conditions, being isolated from several ecological niches including milk, fruit, cereal crops, vegetables, bee bread andfresh meat [28]. As with other LABs, *L. plantarum* is a common host in the gastrointestinal tracts of insects, fish and mammals, including humans [28,29,30]. The metabolic characteristics that are intrinsic of *L. plantarum* are the reason for their versatility and success in industrial applications, not only as a starter culture but also as a probiotic [31,32,33]. Furthermore, some strains of *L. plantarum* are known to produce various natural antimicrobial compounds [34,35], GABA and EPS [36,37,38] and to exhibit antioxidant [39,40] and β-glucosidase activity [41,42]. *L. plantarum* has the Qualified Presumption of Safety (QPS) status from the European Food Safety Authority (EFSA) and Generally Recognized As Safe (GRAS) status from the US Food and Drug Administration (US FDA) [43]. Moreover, they are microorganisms with a documented history of food use [44,45,46]. 

While a considerable number of well-characterized probiotic strains are available worldwide, research into novel strains with specific properties is still of major appeal. This is because even microorganisms within the same species show differential phenotypic characteristics [47,48]. On the basis of this scientific evidence, this work aims to evaluate some bio-functional properties of five *L. plantarum* strains, isolated from bee bread and partially characterized in previous studies [49,50]. In addition to antimicrobial, antioxidant and β-glucosidase activities, the ability of these LABs to produce EPS and GABA were evaluated. The main objective was to evaluate a possible candidacy of these *L. plantarum* strains as dietary supplements in the human diet and/or as microbial cultures for the production of functional food.

## 2. Materials and Methods

### 2.1. Lactiplantibacillus plantarum Cultures

In this investigation, five *L. plantarum* strains (LP 8, LP 25, LP 86, LP 95, and LP 100) isolated from bee bread [49] and belonging to the Food Microbiology Culture Collection of the DiAAA (Department of Agricultural, Environmental and Food Sciences, University of Molise, Campobasso, Italy) were used. 16S rDNA sequences were submitted in GeneBank [51] under accession numbers from OM033651 to OM033655 (accessed on 28 December 2021). *L. plantarum* ATCC 14917, belonging to the American Type Culture Collection (ATCC Manassas, VA, USA), was used as reference strain with proven in vitro probiotic properties [52,53,54].

### 2.2. Antimicrobial Activity

The antimicrobial activity of the *L. plantarum* strains (producers) was evaluated against the following indicator bacteria: *Enterococcus faecalis* ATCC 29212, *Escherichia coli* ATCC 11775, *Proteus mirabilis* ATCC 29906, *Staphylococcus aureus* ATCC 29213 and *Pseudomonas aeruginosa* ATCC 27853, belonging to the American Type Culture Collection (ATCC Manassas, VA, USA). The LABs and the indicator strains were grown at 37 °C in MRS and BHI broth (Oxoid Ltd., Hampshire, UK), respectively.

The *L. plantarum* strains in log phase (10^8^ CFU/mL) were centrifugated (8,000 rpm for 10 min at 4 °C) and filtered (cellulose acetate membrane, pore size 0.22 μm Sigma-Aldrich, Merck KGaA, Darmstadt, GE) to obtain the cell-free supernatant (CFS). The antimicrobial activity of CFS was evaluated according to Iorizzo et al. [50] with some modifications. In brief, 20 mL of BHI soft agar (0.7% agar, *w*/*v*) inoculated with an overnight culture of indicator bacteria (final concentration of about 10^7^ CFU/mL) were poured into Petri plates (Ø 90 mm). Wells of 5.0 mm in diameter were bored into the single plate and 50 μL of CFS, of each producer strain, were placed into different wells. As a control, 50 μL of MRS adjusted to pH 3.8 with hydrochloric acid 1 N (Sigma-Aldrich, Merck KGaA, Darmstadt, GE) was used. After incubation at 37 °C for 48–72 h, the plates were observed and antibacterial activity was reported as the diameter (mm) of the clear zone of inhibition (ZOI) around the inoculated wells [55]. 

### 2.3. Cell Protein Assay

For subsequent analysis (EPS and GABA production; antioxidant activity) the LAB cultures (10^8^ CFU/mL), grown in the specific media reported below, were centrifugated (8000 rpm for 10 min at 4 °C) and filtered (cellulose acetate membrane, pore size 0.22 μm Sigma-Aldrich, Merck KGaA, Darmstadt, GE) to obtain the cell pellet (CP). The CP was washed twice in sterile saline solution (NaCl 0.9%, *w*/*v*), resuspended in NaOH 0.1 M according to Iorizzo et al. [56], and then subjected to three cycles of sonication (Labsonic M; Sartorius Lab Instruments GmbH & Co. KG, Goettingen, GE) at 12 W for 30 s, with 60 s of pause between the cycles to improve cellular lysis. Subsequently, the samples were centrifugated at 13,000 rpm for 5 min at 4 °C, and cell extract supernatants (CES) were used for protein quantification according to Di Martino et al. [57] using a BioSpectrometer (Eppendorf, Hamburg, GE). Total protein concentrations were calculated by means of a calibration curve using Bovine Serum Albumin (BSA) as standard. All chemicals used were supplied by Sigma-Aldrich, Merck KGaA, Darmstadt, GE.

### 2.4. β-Glucosidase Activity

For the assessment of β-glucosidase activity, the tested LABs were cultured in modified MRS (MRS_m_) according to Ávila et al. [58]. The bacterial cultures (10 mL, 10^8^ CFU/mL) were centrifugated (8000 rpm for 10 min at 4 °C) to obtain the cell pellet (CP_GA_); they were washed in sterile saline solution (NaCl 0.9%, *w*/*v*) and resuspended in isotonic Phosphate Buffer Saline (PBS) pH 7.4. The β-glucosidase activity of the *L. plantarum* strains was performed as described by Ávila et al. [58], with some modification. Briefly, β-glucosidase reactions were performed mixing 50 μL of 20 mM *p*-nitrophenyl-β-D-glucopyranoside (*p*-NPG) in PBS pH 6.5, with 50 μL of bacterial suspension (OD_600_ 0.5) and bringing the final volume of the solution to 1 mL with PBS. Next, 200 μL of the mix were placed in a microplate well and read at 405 nm in kinetic mode with a microplate reader (Multiskan™ FC Microplate Photometer, Thermo Fisher Scientific, Waltham, MA, USA) for six hours. Enzyme activity was expressed as the amount of β-glucosidase that released 1 nmol of *p*-nitrophenol (*p*-NP) per unit volume per minute: *p*-NP/(min mL). A calibration curve was obtained using *p*-nitrophenol as standard. The values of β-glucosidase activity were expressed as the ratio of enzyme units. All chemicals used were supplied by Sigma-Aldrich, Merck KGaA, Darmstadt, GE, and the tests were conducted in triplicate.

### 2.5. Exopolysaccharide (EPS) Production

For EPS production, LABs were grown in MRS_m_. Over 48 h at 37 °C, the cultures (10^8^ CFU/mL) were centrifugated (8000 rpm for 10 min at 4 °C) to obtain the cell-pellet (CP_EPS_) and the cell-free supernatant (CFS_EPS_).

The CP_EPS_ and CFS_EPS_ were used for the determination of EPS-b (bound exopolysaccharides) and EPS-r (released exopolysaccharides), respectively, as described by Tallon et al. [59]. EPS quantification was performed by the anthrone-sulfuric acid colorimetric assay as reported by Laurentin et al. [60]. The culture medium without bacterial inoculum was used as control. The EPS-r values are expressed as μmol glucose Eq./mL, while the absolute concentrations of EPS-b* are expressed as nmol glucose Eq./mL. Instead, EPS-b values related to cellular protein content (EPS-b**) were expressed as the ratio between nmol glucose Eq. and cell proteins (μg BSA Eq.). The cell proteins were determined as described in Section 2.3.

The tests were conducted in triplicate. All chemicals used were supplied by Sigma-Aldrich, Merck KGaA, Darmstadt, GE.

### 2.6. Antioxidant Activity (ABTS Assay)

LAB cultures (10 mL) in MRS_m_, over 48 h at 37 °C, were centrifugated (8000 rpm for 10 min at 4 °C) at their log phase (10^8^ CFU/mL) to obtain the cell pellet (CP_AA_). The CP_AA_ were washed twice in sterile saline (NaCl 0.9%, *w*/*v*), resuspended in cold pure methanol (1 mL), and subjected to three cycles of sonication (Labsonic M; Sartorius Lab Instruments GmbH & Co. KG, Goettingen, GE at 12 W for 30 s, with 60 s of pause between the cycles to improve cellular lysis. Subsequently, the samples were centrifugated at 13,000 rpm for 5 min at 4 °C, and cell extract supernatants (CES_AA_) were used to evaluate total antioxidant activity (TAA), using the 2,2 azino-bis 3-ethylbenzothiazoline-6-sulfonic acid (ABTS+) radical cation assay according to Re et al. [61], with some modifications. Briefly, ABTS was dissolved in pure methanol to a concentration of 7 mM. ABTS radical cations (ABTS+) were produced by reacting the ABTS methanol solution with 2.45 mM potassium persulfate (final concentration) and allowing the mixture to stand in the dark at room temperature for 24 h before use. The ABTS·+ solution was diluted with cold pure methanol to an optical density (OD) of 0.700 at 745 nm. Then, 100 μL of CES_AA_ were mixed with 900 μL of the ABTS·+ solution. The OD was measured at 745 nm after 6 min in the dark at room temperature using a BioSpectrometer (Eppendorf, Hamburg, GE). Trolox was used as the standard for the calibration curve.

The antioxidant activity was expressed as the ratio (*w*/*w*) between μg/mL Trolox and mg/mL of cell proteins (BSA Eq.) [62]. The cell proteins were determined as described in paragraph 2.3. All the chemical compounds used were supplied by Sigma-Aldrich, Merck KGaA Darmstadt, GE and the tests were conducted in triplicate.

### 2.7. γ-Aminobutyric Acid (GABA) Production

For the GABA production assay, the *L. plantarum* strains were grown at 37 °C in LFM medium, whose composition is reported in Table S1 (Supplementary Material). The broth cultures (10 mL; 10^6^ CFU/mL) were centrifugated to obtain the cell pellet (CP_LFM_). The CP_LFM_ were washed twice in sterile saline solution (NaCl 0.9%, *w*/*v*), resuspended in 600 μL of ethanol/water (40/60, *v*/*v*), and then subjected to three cycles of sonication (Labsonic M; Sartorius Lab Instruments GmbH & Co. KG, Goettingen, GE Sartorius Lab Instruments GmbH & Co. KG, Goettingen, GE) at 12 W for 30 s, with 60 s of pause between the cycles to improve cellular lysis. Subsequently, the samples were centrifugated at 13,000 rpm for 5 min at 4 °C and cell extract supernatants (CES_LFM_) were used for GABA quantification via High Performance Liquid Chromatography (HPLC) using a Hewlett Packard 1100 (Agilent, Santa Clara, CA, USA) equipped with Degasser G1322A, Binary Pump G1312A, Autosampler ALS G1313A, Thermostatted Column Compartment G1316A, DAD Detector G1315B and Fluorescence Detector G1321A.

For the automatic pre-column derivatization, 20 μL of CES_LFM_ were added to 40 μL of *o*-Phthalaldehyde (OPA) reagent (Agilent, Santa Clara, CA, USA). Subsequently, 30 μL of OPA-derivatives mix were injected into a reverse-phase column (ZORBAX Eclipse Plus C18, 4.6 × 250 mm 5 μm; Agilent, Santa Clara, CA, USA), protected with SecurityGuard Phenomenex (Torrance, CA, USA). The elution was performed at a flow rate of 0.85 mL/min at 27 °C using a solution of sodium acetate buffer 50 mM + tetrahydrofuran 0.3% + methanol 20% pH 6.4 and methanol 100%, as described by Carillo et al. [63]. The fluorescence of OPA derivatives was detected at 340 nm excitation and 450 nm emission wavelengths. The GABA identification and quantification were carried out using HP ChemStation software ver. A.06.03 (Agilent, Santa Clara, CA, USA), compared to retention times and internal peak area of reference standards. The data were expressed as ratio between pmol of GABA and cell proteins content (μg BSA Eq.). The cell proteins were determined as described in paragraph 2.3. All chemicals standards used were supplied by Sigma-Aldrich, Merck KGaA, Darmstadt, GE and the tests were conducted in triplicate.

### 2.8. Statistical Analysis 

Experiments were performed in triplicate (*n* = 3) and all data are expressed as the mean ± standard deviation (SD). Statistical analysis was performed by analysis of variance (ANOVA) followed by Tuckey’s multiple comparisons. Statistical significance was set to *p*-values < 0.05. The software SPSS Statistics 21 (IBM Corp, Armonk, NY, USA) was used for the analysis.

## 3. Results

### 3.1. Antimicrobial Activity

**Figure 1:**
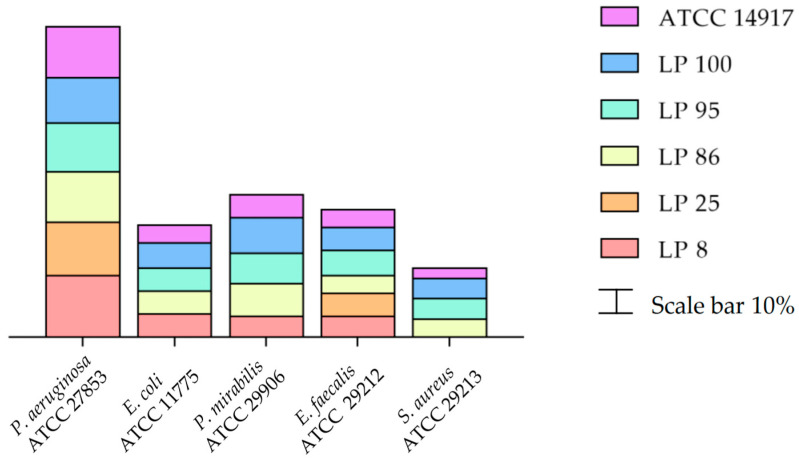
Antimicrobial activity of the *L. plantarum* strains CFS against different indicator bacteria expressed as inhibition rate (%).

The numerical data of the antimicrobial activity, estimated as clear zone of inhibition ZOI (mm), exhibited by LABs against various indicator bacteria are presented in Table S2 (Supplementary Material). In Figure 1, the results are shown as inhibition rate (%). More specifically, no antimicrobial activity was observed when MRS pH 3.8 was used, while the six tested *L. plantarum* strains presented different inhibitory activities against the five indicator bacteria. The cell free supernatant (CFS) of all six *L. plantarum* strains inhibited the development of *P. aeruginosa* and *E. faecalis*. With the exception of LP 25, the other LAB strains were effective against *E. coli* and *P. mirabilis*. The *L. plantarum* LP 86, LP 95, LP 100 and ATCC 14917 strains were effective in counteracting the growth of *S. aureus*. The highest antagonistic activity of the tested *L. plantarum* strains was against *P. aeruginosa* with a ZOI between 18 (LP 100) and 24 mm (LP 8). 

### 3.2. EPS Production

The values of EPS production were reported in Table 1. In detail, the EPS-r values ranged from 2.84 (LP 25) to 3.43 μmol glucose Eq./mL (LP 8), while the values of EPS-b* varied from 38.95 (LP 86) to 223.42 nmol glucose Eq./mL (LP8). Lastly, about the EPS-b** content, the range of values was between a minimum of 0.016 (LP 86) and a maximum of 0.069 (LP 95) nmol glucose Eq./μg BSA Eq. (bovine serum albumin).

### 3.3. ABTS Antioxidant Activity

The ABTS radical-scavenging assay has been used to assess the ability of metabolic compounds produced by the different *L. plantarum* strains to act as free radical scavengers or hydrogen donors. The Trolox equivalent antioxidant capacity is expressed with respect to cellular protein content. As reported in Table 2, antioxidant activity values are significantly different among the tested strains. In detail, the strains LP 95 and LP 100 exhibited the highest values, which amounted to 14.23 and 15.84 μg Trolox Eq./mg BSA Eq., respectively, while the lowest value has been found for strain LP 86 (3.89 μg Trolox Eq./mg BSA Eq.).

### 3.4. β-Glucosidase Activity

The enzymatic activity exhibited by the bacterial cultures was assessed as the release of *p*-NP from the *p*-NPG substrate in the time unit. As reported in Table 2, all tested strains showed β-glucosidase activity, which ranged from 0.26 to 0.45 nmol *p*-NP/(min mL). The data show significant differences in this specific enzymatic activity among the strains. The minimum values were recorded for LP 100 and ATCC 14917 strains, whereas the highest value corresponded to *L. plantarum* LP 95.

### 3.5. GABA Production

**Figure 2:**
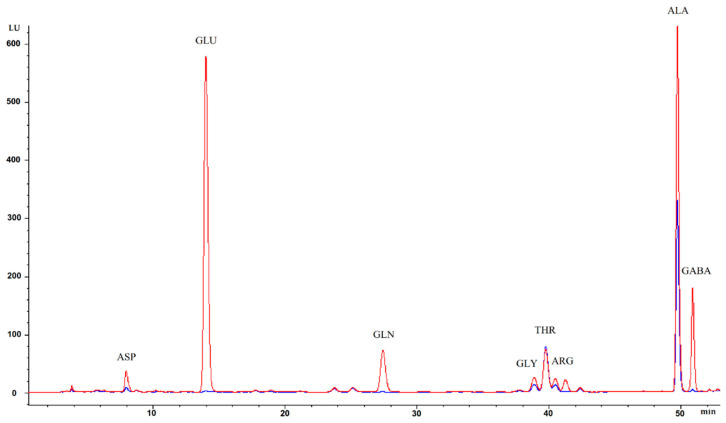
GABA production from *L. plantarum* LP 95 in LFM medium with (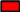) and without (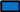) L-glutamic acid (GLU).

**Figure 3:**
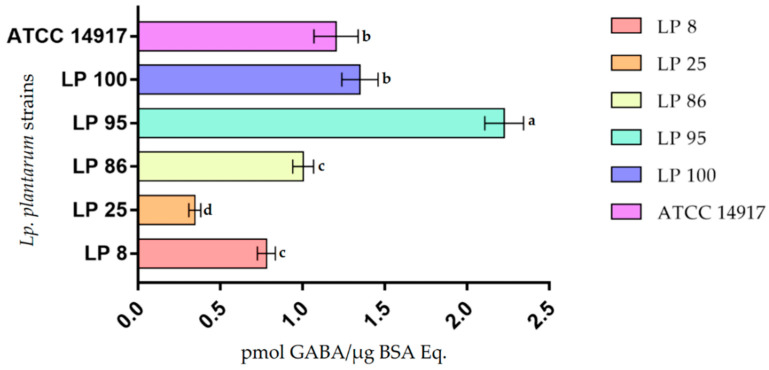
GABA amounts synthesized from *L. plantarum* strains grown in LFM medium with L-glutamic acid (GLU) (100 μM). All data are expressed as mean ± standard deviation (*n* = 3). Different lowercase letters (a–d) indicate significant differences (*p* < 0.05).

The biosynthetic ability of *L. plantarum* strains to produce GABA was assessed in LFM medium with and without L-glutamic acid (GLU). GABA contents were determined by injecting an aliquot of the cell extract supernatants (CES_LFM_) via HPLC after precolumn OPA derivatization, and the amounts were calculated as the internal peak area. Representative chromatograms are shown in Figure 2. The results, reported in Table S3 and summarized in Figure 3, were expressed as the ratio between the pmol of GABA and the cell proteins content (μg BSA Eq.) The GABA-specific contents in the CES_LFM_ obtained from the six *L. plantarum* strains examined showed significant differences as shown in Figure 3. Values varied within a range of 0.345 (LP 25) and 2.27 μg Trolox Eq./mg BSA Eq. (LP 95). 

## 4. Discussion

The characterization of microorganisms beneficial for food preservation and the prevention of pathogen growth is mainly based on their ability to produce bioactive compounds that have antimicrobial activity.

In our investigation the reference strain ATCC 14917 and the five *L. plantarum* isolated by us from bee bread [49,50] were able to limit the growth of indicator bacteria, but with varying degrees of intensity. The mechanisms underlying the inhibitory capacity of LABs are attributable to multiple mechanisms of action, such as individual compounds that may cause membrane destabilization (such as fatty acids or peptides), H^+^ gradient interference (such as organic acids or peptides), or enzyme inhibition (such as hydroxy acids) [64]. Moreover, there may be some synergistic and/or additive effects that involve various compounds [65].

Since no effect on the growth of indicator bacteria was observed in our study using MRS pH 3.8, the inhibition of the LABs against pathogens cannot be ascribed to a simple consequence of the lowering of pH due to the bacterial metabolism of the producer strains. Antimicrobial activity, therefore, was most likely caused by the production of organic acids, protein compounds or other antimicrobial metabolites contained in CFS, which need to be further investigated.

The LAB food-grade EPSs have earned considerable attention for their structural and sensory properties in the food industry, primarily for dairy products and more recently for the improvement of cereal-based products [37,66,67,68,69,70,71,72]. Furthermore, EPSs have also gained major relevance in pharmacological and nutraceutical applications, as they can serve as immunomodulatory, antioxidant, anti-cancer, cholesterol-lowering, antimicrobial and prebiotic agents [73,74,75,76,77]. Microbial EPSs are not permanently anchored to the microbial cell surface and exist in two forms based on their localization: cell-bound EPSs, which are tightly adhered to the bacterial surface (bound exopolysaccharides; EPS-b), and EPSs that are released into the surrounding medium (released exopolysaccharides; EPS-r). In our research, we quantitatively determined the two exopolysaccharide matrices and showed the ability of the tested *L. plantarum* strains to produce these compounds with significant differences in EPS-b production. 

Our results confirm that several *L. plantarum* strains are good EPS producers [14,37,78]. In particular, the strains LP 95 and LP 100 produced quantities significantly higher compared to the other strains. The rheological and prebiotic properties of the EPS produced by LAB [66] make it for possible technological applications in the food industry of the *L. plantarum* LP 95 and LP 100 strains. 

Some LAB strains possess both enzymatic and non-enzymatic antioxidant activity, which reduces the risk of reactive oxygen species (ROS) accumulation during food ingestion, resulting in a decrease in oxidative stress [79,80,81,82]. Over the last decade, considerable attention has been drawn to the use of LAB as natural antioxidants in the food industry [83]. Apart from some cellular constituents (exopolysaccharides, peptidoglycan, lipoteichoic acid and proteins) LABs are known to have complex antioxidant activity, and different strains operate different mechanisms: chelation of toxic ions (Fe^2+^ and Cu^2+^), synthesis of antioxidant compounds (e.g., glutathione, butyrate, folate) and activation of enzymatic pathways for the scavenging of free radicals [39,84,85,86,87]. It has also been reported that the antioxidant activity of LABs may be linked to some proteolytic-derived peptides and may vary depending on the species and on the strain [83].

Whereas in other studies, the antioxidant activity of various *L. plantarum* strains is associated with EPS [85,87,88].

Tang et al. [81] reported that CFS from *L. plantarum* MA2 exhibited strong reducing capacity, lipid peroxidation inhibition, Fe^2+^-chelating and free radical scavenging activities. Moreover, the cell homogenate revealed glutathione peroxidase activity and superoxide dismutase activity.

In our work, we evaluated the antioxidant activity by means of the ABTS assay, which is considered one of the most sensitive procedures [89] and a valid method for the assessment of the antioxidant activity of both hydrophilic and lipophilic extracts [90].

All five strains of *L. plantarum* exhibited antioxidant activity, and the mechanism or molecules involved in this behavior require more detailed study.

Over recent years, the pharmaceutical and food industries’ research has strongly focused on the development of dietary supplements and fermented foods enriched with GABA for the various health benefits of this amino acid, including neurostimulation and cardio protection [10,11]. GABA-producing LABs may provide new opportunities for naturally fermented functional food products [16]. LABs synthesize GABA via the GAD pathway, in which the GAD protein localized in the cytoplasm is the key enzyme. Strains with the *gad* gene are therefore able to synthesize GABA. The first step in the GAD pathway is carried out by a GLU/GABA antiporter, encoded by a *gadC* gene. This antiporter pumps the GLU precursor into the microorganism [10]. LABs have strain-specific GABA synthesis capacities and several environmental factors, such as pH and temperature, impact the expression of the *gad* gene [91,92]. *L. plantarum* is one of the most widely distributed and common bacteria occurring in fermented foods and therefore it would be valuable to achieve effective GABA production using selected strains from this species.

Our results showed that the tested *L. plantarum* strains are able to synthesize GABA, confirming that bacteria belonging to this species are able to produce this amino acid [93,94]. To assess the ability to produce GABA, we used the medium LFM (Table S1) with a defined centesimal composition to exclude interference from undefined substances in the MRS medium based on complex matrices (e.g., yeast extract and meat extract).

LABs with the ability to synthesize GABA can offer new opportunities in the design of functional foods. However, further investigations are needed to optimize the fermentation conditions and to improve the production of this non-proteic amino acid [11,95,96].

Isoflavones are plant-based polyphenolic compounds, predominantly found in glycosidic forms that can be hydrolyzed by intestinal and bacterial β-glucosidases to release aglycones, which are subsequently absorbed in the large intestine [20,41].

β-glucosidase LAB producers have a major role in the release of free flavonoids from their glycosylate precursors found in fruits and other plant tissues and enhance the bioavailability of these health-promoting plant metabolites [97]. 

In our study, the selected *L. plantarum* strains exhibited β-glucosidase activity without significant differences among them. As a result of this specific enzymatic activity, it would therefore be interesting to use them as starter cultures in future applications to optimize the organoleptic, technological and nutritional properties of fermented foods (e.g., soy-derived foods or vegetable beverages) [12,97].

## 5. Conclusions

This in vitro study allowed us to screen some *L. plantarum* strains possessing good potential for further studies on their probiotic and functional properties.

We found a large heterogeneity related to examined properties confirming that these are strain-specific.

Therefore, our results suggest that a careful selection within a given species is of utmost importance to identify appropriate strains for specific biotechnological applications. In particular, the evaluation of our results shows that *L. plantarum* LP 95 was the best-performing strain and that could be a candidate as new a probiotic in the para-pharmaceutical sector and as food microbial culture to improve some functional characteristics of specific products. 

Clearly, we consider this study a preliminary selection and further in vitro and in vivo investigations are needed for the use of this LAB in human diet.

## Figures and Tables

**Table 1:** EPS-released (EPS-r), EPS-bound (EPS-b) and EPS-bound related to cell proteins (EPS-b**). All values are expressed as mean ± standard deviation (*n* = 3). Different lowercase letters (a–d) in each row indicate significant differences (*p* < 0.05).

*L. plantarum* Strains						
EPS Fractions	LP 8	LP 25	LP 86	LP 95	LP 100	ATCC 14917
**EPS-r**	3.43 ± 0.08 ^a^	2.84 ± 0.15 ^b^	3.29 ± 0.20 ^a^	3.06 ± 0.17 ^a^	3.03 ± 0.18 ^a^	3.16 ± 0.13 ^a^
EPS-b*	135.91 ± 5.67 ^c^	136.23 ± 5.08 ^c^	38.95 ± 1.91 ^d^	223.42 ± 7.90 ^a^	160.96 ± 7.94 ^b^	51.45 ± 2.74 ^d^
EPS-b**	0.048 ± 0.007 ^b^	0.046 ± 0.005 ^b^	0.016 ± 0.004 ^c^	0.069 ± 0.011 ^a^	0.050 ± 0.004 ^b^	0.026 ± 0.006 ^c^

The data are expressed as follows. EPS-r: μmol glucose Eq./mL; EPS-b*: nmol glucose Eq./mL; EPS-b**: nmol glucose Eq./μg BSA Eq.

**Table 2:** Antioxidant and β-glucosidase activities of *L. plantarum* strains. All values are expressed as mean ± standard deviation (*n* = 3). Different lowercase letters (a–c) in each row indicate significant differences (*p* < 0.05).

*L. plantarum* Strains						
	LP 8	LP 25	LP 86	LP 95	LP 100	ATCC 14917
* Antioxidant **activity**	6.46 ± 0.65 ^c^	6.51 ± 0.38 ^c^	3.89 ± 0.34 ^d^	14.23 ± 1.60 ^a^	15.84 ± 0.80 ^a^	13.35 ± 0.26 ^b^
** β **-glucosidase** **activity**	0.30 ± 0.06 ^a^	0.39 ± 0.07 ^a^	0.38 ± 0.08 ^a^	0.45 ± 0.03 ^a^	0.26 ± 0.08 ^b^	0.26 ± 0.08 ^b^

* Expressed as μg Trolox Eq./mg BSA Eq.; ** Expressed as nmol *p*-Nitrophenol/(min mL).

## Data Availability

The data presented in this study are available in the Supplementary Materials.

## Supplementary Materials

The following supporting information can be downloaded at: https://www.mdpi.com/article/10.3390/cimb44050158/s1, Table S1: LFM medium composition; Table S2: Antimicrobial activity by cell-free supernatant of the different *L. plantarum* strains against indicator bacteria; Table S3: GABA intracellular contents of the different *L. plantarum* strains.
