# Supplementary material for: In Vitro Assessment of Bio-Functional Properties from Lactiplantibacillus plantarum Strains
Source: Curr Issues Mol Biol. 2022 May 19;44(5):2321–34. doi: 10.3390/cimb44050158 (PMC9164048; doi:10.3390/cimb44050158)
Supplement: Supplementary file 1 [file cimb-44-00158-s001.zip › cimb-1716990-supplementary.pdf]

current issues in molecular biology

**Table S1.** LFM medium composition. Yeast Nitrogen Base (Sigma-Aldrich, Merck KGaA, Darmstadt, GE) supplemented with:.

| | |
|---|---|
| Pyridoxal-5'-phosphate | 40 mg/L |
| D-Glucose | 20g/L |
| L-Cysteine | 100 µM |
| L-Phenylalanine | 100 µM |
| L-Histidine | 100 µM |
| L-Glycine | 100 µM |
| L-Glutamic acid | 100 µM |

**Table S2.** Antimicrobial activity at 37 °C by cell-free supernatant (CFS) from the five *Lp. plantarum* strains against different indicator bacteria. The data (mean ± SD; n = 3) are expressed as zone of inhibition-ZOI (mm). Different lowercase letters (a–c) in each row indicate significant differences ($p < 0.05$).

| **Indicator bacteria** | ***Lp. plantarum* strains** | | | | | |
|---|---|---|---|---|---|---|
| | **LP 8** | **LP 25** | **LP 86** | **LP 95** | **LP 100** | **ATCC 14917** |
| *P. aeruginosa ATCC 27853* | 24.0 ± 3.0[a] | 21.0 ± 1.0[a] | 20.0 ± 2.0[a] | 19.0 ± 2.0[a] | 18.0 ± 3.0[a] | 20.0 ± 2.0[a] |
| *E. coli ATCC 11775* | 9.0 ± 3.0[a] | 0.0 ± 0.0[b] | 9.0 ± 2.0[a] | 9.0 ± 1.0[a] | 10.0 ± 1.0[a] | 7.0 ± 3.0[a] |
| *P. mirabilis ATCC 29906* | 8.0 ± 2.0[b] | 0.0 ± 0.0[c] | 13.0 ± 2.0[a] | 12.0 ± 3.0[a] | 14.0 ± 1.0[a] | 9.0 ± 3.0[a] |
| *E. faecalis ATCC 29212* | 8.0 ± 1.0[a] | 9.0 ± 1.0[a] | 7.0 ± 2.0[a] | 10.0 ± 1.0[a] | 9.0 ± 3.0[a] | 7.0 ± 2.0[a] |
| *S. aureus ATCC 29213* | 0.0 ± 0.0[c] | 0.0 ± 0.0[c] | 7.0 ± 1.0[a] | 8.0 ± 1.0[a] | 8.0 ± 2.0[a] | 4.0 ± 2.0[b] |

**Table S3.** GABA production (pmol GABA/µg BSA Eq.) from the five *Lp. plantarum* strains grown in LFM medium with and without L-glutamic acid (GLU). All values are expressed as mean ± standard deviation (n = 3). Different lowercase letters (a–d) in each row indicate significant differences ($p < 0.05$).

| | GABA production of *Lp. plantarum* strains | | | | | |
|---|---|---|---|---|---|---|
| **LFM medium** | **LP 8** | **LP 25** | **LP 86** | **LP 95** | **LP 100** | **ATCC 14917** |
| without GLU | 0.068±0.011[b] | 0.060±0.011[b] | 0.047±0.008[b] | 0.055±0.008[b] | 0.088±0.006[a] | 0.116±0.031[a] |
| with GLU | 0.781±0.055[c] | 0.345±0.026[d] | 1.004±0.063[c] | 2.227±0.118[a] | 1.350±0.111[b] | 1.204±0.134[b] |
